# Iodide of Iron

**Published:** 1846-02

**Authors:** 


*Iodide of Iron.*—A writer in the Boston Medical and Surgical Journal, April 30th, 1845, recommends the following as an easy method of preparing a very perfect syrup of the Iodide of Iron:

"Take of Iodide 200 grains, iron wire half an ounce, white sugar in powder four ounces and a half, distilled water one fluid ounce. Agitate the iodide, wire, and water together briskly in a strong two ounce vial with a glass stopper, until the froth becomes white. Pour the fluid immediately into the sugar in a glass or wedgewood mortar. Triturate a few minutes, and then add distilled water enough to make six fluid ounces. Twelve minims of this syrup contain one grain of iodide of iron."

In the same communication the following simple method of preparing pills of the iodide of iron, as recommended by Dr. Christison, is quoted:

"Take of iodide 129 grains, iron wire about the thickness of a thin quill, half an ounce, distilled water 75 minims. Agitate them briskly together in a strong ounce vial, provided with a well-fitted glass stopper, until the froth becomes white, which will happen in less than ten minutes. Pour the liquid upon two drachms of finely powdered loaf sugar in a little mortar, and triturate immediately and briskly for a few minutes; add gradually a mixture of the following powders, viz: Liquorice powder half an ounce, powder of gum arabic a drachm and a half, and flour one drachm. Divide into 144 pills. Each pill contains about one grain of iodide of iron."

The iodide of iron is becoming a favorite remedy with the profession. The two constituents are adapted to meet indications which are often presented together, and each element seems to derive advantage from the combination. The tendency to decomposition, however, is a serious difficulty in the way of its administration in a simple state. The presence of saccharine matter is found to be necessary to prevent this difficulty, and the two recipes which are given above, while they serve this object, have the merit of simplicity.—*Ibid.*

